# How Reliable Is the Electrochemical Readout of MIP Sensors?

**DOI:** 10.3390/s20092677

**Published:** 2020-05-08

**Authors:** Aysu Yarman, Frieder W. Scheller

**Affiliations:** Institute of Biochemistry and Biology, University of Potsdam, Karl-Liebknecht-Strasse 24-25, 14476 Potsdam, Germany

**Keywords:** molecularly imprinted polymers, electropolymerization, direct electron transfer, catalysis, redox marker, gate effect

## Abstract

Electrochemical methods offer the simple characterization of the synthesis of molecularly imprinted polymers (MIPs) and the readouts of target binding. The binding of electroinactive analytes can be detected indirectly by their modulating effect on the diffusional permeability of a redox marker through thin MIP films. However, this process generates an overall signal, which may include nonspecific interactions with the nonimprinted surface and adsorption at the electrode surface in addition to (specific) binding to the cavities. Redox-active low-molecular-weight targets and metalloproteins enable a more specific direct quantification of their binding to MIPs by measuring the faradaic current. The in situ characterization of enzymes, MIP-based mimics of redox enzymes or enzyme-labeled targets, is based on the indication of an electroactive product. This approach allows the determination of both the activity of the bio(mimetic) catalyst and of the substrate concentration.

## 1. Introduction

Over the past decades, increasing attention has been paid to the fast, selective and cost-effective detection and determination of analytes in many areas, including clinical diagnostics, pharmaceutical and environmental analysis, food control and security. Well-established laboratory-based (bio)analytical methods achieved great breakthroughs due to the highly specific interactions involved in most biological processes, e.g., the antigen–antibody interaction, substrate conversion by the action of enzymes and the sequence-specific hybridization of nucleic acids [1].

**Figure 1:**
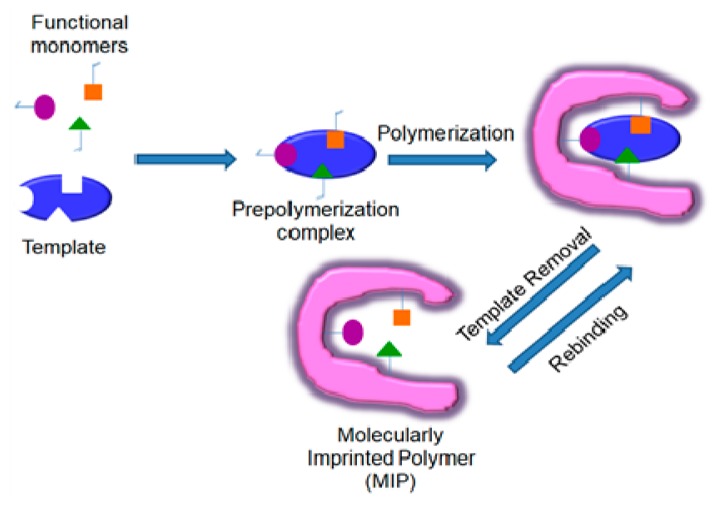
Schematic representation of MIP preparation.

Nevertheless, biochemical reagents also have some drawbacks, such as stability under harsh conditions (high temperature, organic solvents, limited pH range), reusability and animal usage in preparation (antibodies). Starting from supramolecular chemistry, molecularly imprinted polymers (MIPs) have been created, which potentially overcome these drawbacks [2,3,4,5,6,7,8]. They are prepared by polymerizing functional monomers in the presence of a target analyte (template). The subsequent removal of the template from the polymer results in the formation of cavities with a molecular memory mirroring the size and shape of the template (Figure 1). MIPs mimic the binding sites of antibodies by substituting the amino acid scaffold for synthetic polymers. Furthermore, catalytically active MIPs containing metal ions or prosthetic groups of oxidoreductases have been developed, which exhibit enzyme-like activity towards substrates [9,10]. The polymer scaffold of the MIP provides specificity by substrate binding to the cavities while the metal complex is the reactive center. The performance of MIPs has also been markedly enhanced by incorporating nanomaterials [11,12] and, as a new trend, by integration in metal organic frameworks (MOFs) [13,14].

For a good analytical performance of the sensor, the MIP should be placed immediately on the surface of the electrode. Two different procedures for the preparation of MIP sensors have been described in the literature [15].

(i) In the first procedure, the MIP is separately synthesized and then immobilized on the transducer surface. In the past, MIPs were most frequently synthesized using bulk polymerization. As a result, monolithic materials are produced, which are then ground into smaller particles. The major disadvantage of bulk polymerization is the bad accessibility and inhomogeneity of the binding pockets, which leads to a longer template removal time and slow rebinding. To overcome these problems, different forms of MIPs, such as micro- or nanobeads, nanoparticles or nanospheres have been prepared [16,17,18,19].

For the integration of MIPs in the body of the sensor, different methods have been used [15,20,21]. The simplest approach is drop coating [22]. Furthermore, spin coating or spray coating have been applied [23]. Grafting is another approach used for the incorporation of the MIPs [24]. In addition to the described approaches, MIPs can also be integrated via the preparation of composite membranes or layer-by-layer assembly [15,20].

(ii) In the second procedure, the MIP-based recognition layer is directly formed on the transducer. In addition to the formation of an MIP layer by self-polymerization [25] and the microcontact imprinting of a soft polymer cover layer [26], electropolymerization is the most straightforward way to prepare MIPs directly on the conductive surface of a transducer, e.g., on an electrode, QCM or SPR chip [15,20,27]. An advantage of electrosynthesis is that the film thickness can be adjusted by varying the charge passed during the polymerization. The selection of the solvent and supporting electrolyte and the regime of potential applications influence the morphology of the polymer layer [20,28]. Furthermore, the application of potential pulses is a simple method for removing the template after the MIP synthesis.

Molecular recognition by MIPs has been coupled in biomimetic sensors with a whole arsenal of transducers [20,28,29,30,31,32,33,34,35,36]. Among them, electrochemical and optical techniques clearly dominate [31,37]. In addition, piezoelectric [38,39,40], thermal [41,42] and micromechanical [43,44] transducers have been applied in MIP sensors. All steps of MIP synthesis, and of the measurement, can be analyzed by methods directly indicating the presence of the target molecule in the MIP layer, or by indirect methods evaluating the change in the signal of a marker [1,31,45]. The direct detection of the template molecules by the redox conversion at an electrode [46], intrinsic fluorescence of the target or of a label [47], Raman and FTIR spectroscopy [48] or surface-enhanced infrared absorption (SEIRA) spectroscopy [49] specifically indicate the presence of the template in the MIP during the removal and rebinding of the target. In contrast, surface plasmon resonance (SPR), quartz crystal microbalance (QCM), and capacitor- or thermistor-based sensing systems reflect specific binding, nonspecific adsorption to the polymer surface and other types of changes in the chemical environment [1,33,42,50,51]. 

Another important aspect is the assay format. The determination of thermodynamic parameters, e.g., the binding constant, requires the generation of the measuring signal under equilibrium conditions, i.e., the affinity sensor is in a target-containing solution. Only for binder–target pairs, with a very low rate of dissociation, can the measurement be performed in a target-free solution. On the other hand, for “dynamic” systems, the dissociation rate can be determined by the decay of the signal after the removal of the target, which is an approach frequently used for SPR and QCM sensors [40,52].

In this review, the focus will be on the electrochemical readout of MIP sensors.

## 2. Electrochemical Readout

The electrochemical readout of biosensors started in 1962 with the first glucose sensor by Leland Clark [53]. Enzyme electrodes allow the indirect measurement of an electroinactive analyte by indicating the concentration change of an electroactive reaction partner, e.g., oxygen, hydrogen peroxide, a redox marker or the change of the pH. Electrochemical methods have been successfully transferred to immunosensors [54] and nucleic acid arrays [55]. Electrochemical biomimetic sensors based on MIPs or aptamers have also been developed [56]. 

Among the electrochemical approaches, a comparably low number of potentiometric MIP sensors, capacitors or field effect transistors have been presented, while voltammetric methods are widely used [28,33,57,58,59]. The potential window of voltammetric sensors is restricted by anodic oxygen evolution and cathodic hydrogen generation. This potential region is larger for carbon-based electrodes as compared with noble metal electrodes. The measuring potential of the electrode is decisive for the selectivity of the sensor. Any electroactive substance with a lower redox potential is electrochemically converted, thus contributing to the electrode signal. Pulse methods like differential pulse voltammetry (DPV) and square wave voltammetry (SWV) are effective methods to suppress electrochemical interferences and to increase sensitivity by eliminating the charging current. Additionally, a large spectrum of nanomaterials, including nanoparticles, carbon nanotubes and graphene, has been successfully applied to improve the analytical performance of electrochemical sensors, including MIPs [11,12].

**Figure 2:**
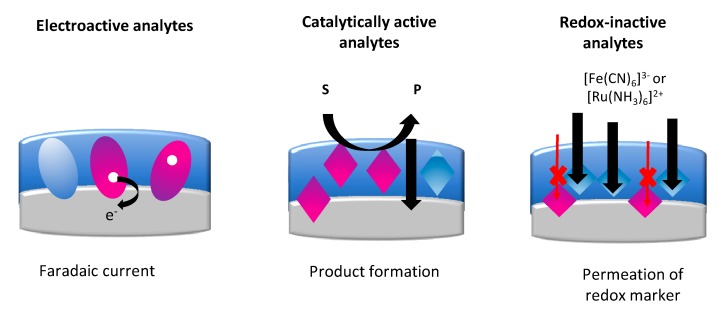
The main approaches used in electrochemical readouts of MIP-based sensors.

Electrochemical methods are especially appropriate for the direct quantification of redox-active analytes, and for the indication of redox enzymes or enzyme mimics, by measuring the formation of electroactive products. For the measurement of electroinactive analytes by affinity sensors, redox-active labels or enzyme “tracers” have been used to generate an electrochemical signal. As a general approach for all affinity sensors, the modulation of the electrochemical signal of a redox marker has been introduced. These electrochemical approaches have been adapted for the readout of MIP sensors (Figure 2) [1,20,31,37].

(i) When the targets are redox-active, the faradaic current is measured, which is based on the direct electron transfer (DET) between the target and the underlying electrode.
(ii) In the case of enzyme targets, catalytically active MIPs or enzyme-labeled targets, the enzymatic activity of the MIP layer is detected via the generation of a redox-active product at the electrode surface.
(iii) Most of the research covers the flux of a redox marker. The signal modulated by the target binding is detected at the underlying electrode surface.

### 2.1. Electroactive Analytes

The most specific detection of rebinding to the MIP is the electrochemical conversion of the analyte. In this case, the signal originates from the template reaching to the electrode surface. The lack of selectivity may originate from “nonspecific pores” in the polymer layer, but not from the insufficient selectivity of the imprinted sites. The MIP film acts as a “molecular filter” on the electrode surface, which discriminates the constituents of the sample according to the size and shape of the molecules. This “filtering” leads to a marked improvement in the specificity as compared with the bare electrode. However, the partial “blockage” of the electrode surface decreases the sensitivity as compared with the bare electrode. The integration of nanomaterials such as nanoparticles, carbon nanotubes or graphene in the MIP layer increases the active surface area, thus enhancing sensitivity [11].

This measuring principle has been applied to a broad spectrum of low molecular-weight-substances, such as drugs (tamoxifen, paracetamol and L-DOPA [60,61,62]), veterinary drugs [63], hormones [64], chemicals (pesticides [65,66] and mycotoxins [67]) or drugs of abuse [68]. 

As early as 1995, Kriz and Mosbach described an amperometric detection system for morphine based on MIPs [69]. MIP particles were immobilized on a Pt electrode via agarose. The measurement involved two steps. In the first step, morphine was bound to the MIP, resulting in an increase in current. In the next step, after signal stabilization, the electroinactive competitor, codeine, was added to the measuring solution, which caused the release of morphine from the MIP, resulting in a further increase in current due to the oxidation of morphine.

Another frequently applied voltammetric method for the detection and determination of electroactive analytes is differential pulse voltammetry. In 2007, Ozcan and Sahin developed an MIP sensor for the analgesic and antipyretic drug paracetamol [61]. The MIP was prepared by the electropolymerization of pyrrole in the presence of the drug on a graphite electrode. They evaluated the performance of the MIP by means of DPV. The sensor showed two linear regions: 5 µM–0.5 mM and 1.25–4.5 mM. The limit of detection (LOD) was calculated to be 0.79 µM. They also showed that the presence of a two-fold excess of potential interferences like glucose, phenacetin, dopamine, ascorbic acid and phenol did not influence the paracetamol response. 

Furthermore, different nanomaterials were incorporated into the MIP sensors to enhance the signal [11,12]. For example, Li et al. applied Ag/N-doped reduced graphene oxide (Ag/N-RGO) in the MIP sensor for the determination of salbutamol, which is an β2-adrenergic agonist [70]. The MIP was prepared on the Ag/N-RGO-modified glassy carbon electrode (GCE) via electropolymerization. Cyclic voltammetric measurements demonstrated that in a 0.1 mM salbutamol solution, the lowest signal was obtained with bare GCE, whereas Ag/N-RGO-MIP-GCE showed the highest signal. DPV was applied for the quantitative determination. The linear range was found to be 0.03–20 µM with an LOD of 7 nM. 

**Figure 3:**
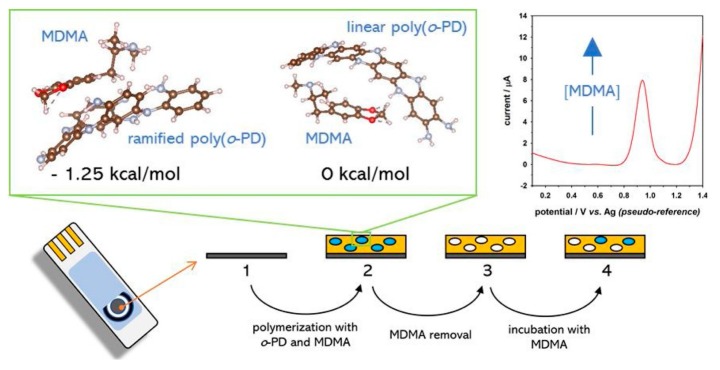
Schema of ecstasy-imprinted polymer on a screen-printed electrode and direct electrochemical detection of ecstasy (MDMA: 3,4-methylenedioxymethamphetamine) binding. Reprinted by permission from [68].

In addition to disk or wire electrodes, screen-printed electrodes have also widely been applied in MIP sensors. Couto et al. have recently presented an MIP sensor for the direct detection of ecstasy (MDMA: 3,4-methylenedioxymethamphetamine), which is one of the most common narcotics (Figure 3) [68]. The sensor was prepared on a screen-printed carbon electrode by electropolymerization in a solution of o-phenylenediamine and MDMA. The binding of MDMA was detected by square wave voltammetry. The sensor exhibited a linear response of up to 0.2 mM with an LOD of 0.79 µM. Moreover, selectivity studies have been performed with structurally similar substances. The selectivity factor, which is the ratio of the MDMA peak current and the interfering substances, has been calculated to be 5.6 and 2.8 for dopamine and tyramine, respectively. They further applied the sensor in biological fluids. 

Moghadam et al. prepared an MIP sensor on a screen-printed carbon electrode for the determination of the antibiotic oxacillin (OXC) [71]. Prior to the electropolymerization of aniline, gold nanourchin and graphene oxide were immobilized on the electrode. The linear response obtained by means of DPV was in the concentration range of 0.7–575 nM and the LOD was determined to be 0.2 nM. 

Moro et al. developed an MIP sensor for the β-lactam antibiotic cefquinome (CFQ) on a multi-walled carbon nanotube-modified graphite screen-printed electrode [63]. The sensor showed a linear response (50 nM–50 µM) only after applying two steps, i.e., incubation in CFQ and measurement in fresh CFQ-free solution. 

Recently, Amatatongchai et al. exploited screen-printing technology on a paper-based system for the detection of serotonin [22]. Nanosized MIP particles were prepared by encapsulating Fe_3_O_4_@Au nanoparticles with silica, which was imprinted by the sol-gel method. These particles were then drop-casted onto the graphite electrode of the paper-based device. Serotonin was quantified by linear sweep voltammetry. The linear range and LOD were determined to be 0.01–1000 µM and 0.002 µM, respectively. Furthermore, the device showed no interference for ascorbic acid, uric acid, dopamine, glucose norepinephrine or ions like Mg^2+^ and Ca^2+^. 

The simultaneous determination of several analytes is of great interest in some areas, such as clinical and pharmaceutical analysis. MIPs have been applied in the simultaneous determination of different analytes as well [72,73,74]. Zheng et al. developed an electrochemical MIP sensor for the direct detection of uric acid and tyrosine [72]. The MIP was prepared on a reduced graphine oxide-modified electrode using a novel monomer of 2-amino-5-mercapto-1,3,4-thiadiazole and a dual template via electropolymerization. DPV was applied to characterize the analytical performance of the sensor. The MIP exhibited two linear regions for uric acid, i.e., 0.01–1 µM and 4–100 µM with an LOD of 0.0032 µM. Two linear regions were also observed for tyrosine, i.e., 0.1–10 µM and 40–400 µM with an LOD of 0.046 µM. In addition, a 50-fold concentration of the potential interferences dopamine, epinephrine, adenine, xanthine, ascorbic acid and glucose had a negligible effect on uric acid and tyrosine sensing by the MIP sensor, whereas with a reduced graphene oxide-modified glassy carbon electrode, pronounced interferences were found. In another work, an MIP sensor for rifampicin (RIF) and isoniazid (INZ) was developed [74]. Prior to the electropolymerization of pyrrole, the glassy carbon electrode was modified with a copper metal organic framework/mesoporous carbon composite. This modification enhanced the sensitivity of the sensor. Adsorptive stripping differential pulse voltammetry showed that the sensor’s response was linearly dependent for both RIF and INZ on the concentration in the range of 0.08–85 μM and the LODs were determined to be 0.28 nM and 0.37 nM for RIF and INZ, respectively. Moreover, simultaneous determinations of the drugs were realized in serum, urine and pharmaceutical dosages as well as in aqueous solutions.

It is known that the anodic oxidation of phenolic substances generates a polymer film, which causes a decrease in sensitivity by the “fouling” of the electrode surface. In order to prevent this adverse effect, the analyte was converted in a preceding enzymatic reaction into a product, which was indicated at a lower potential at the electrode than the polymer formation [75,76]. Yarman and Scheller have applied this approach for an electrochemical MIP sensor for the analgesic drug aminopyrine, which is converted by horseradish peroxidase to aminoantipyrine in a layer on the top of an aminoantipyrine MIP [75]. Therefore, aminoantipyrine was used as the target of the electrosynthesized MIP. The rebinding of aminoantipyrine to the aminoantipyrine-imprinted electropolymer was measured using the oxidation current at 0.5 V. The amperometric current response of the MIP-covered glassy carbon electrode was linearly dependent on the concentration up to 110 µM. The imprinting factor was calculated to be 6.67. The highest signal was observed for the template as compared to ascorbic acid, uric acid and caffeine. Furthermore, an HRP-catalyzed reaction allowed a measurement at a lower potential, i.e., 0 V, which led to the complete elimination of interfering substances.

**Figure 4:**
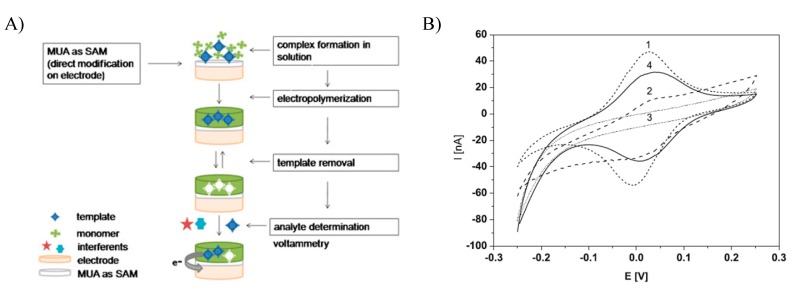
(**A**) Workflow of the preparation of the cytochrome c MIP and (**B**) the electrochemical readout indicating the DET at the MUA-covered Au electrode (1), after electropolymerization (2), after template removal (3) and rebinding (4). Reprinted by permission from [46].

Only a limited number of MIPs for redox enzymes and metalloproteins exploiting DET have been published. This measuring principle is restricted to “extrinsic” redox enzymes with surface-exposed redox centers, which exchange electrons with electrodes without soluble mediators [77]. The first MIP exhibiting DET was developed for the hemeprotein cytochrome c by Scheller’s group [46]. The target protein was pre-adsorbed at a negatively charged self-assembled monolayer (SAM) of mercaptoundecanoic acid (MUA) prior to the polymer deposition (Figure 4). The surface concentration of cytochrome c, which was calculated from cyclic voltammetric measurements, increased linearly up to 4 µM. Furthermore, competition experiments with other proteins (bovine serum albumin, myoglobin and lysozyme) demonstrated that the MIP had preferential binding to its target, cytochrome c. Following this procedure, an MIP was synthesized around a more complex protein, hexameric tyrosine-coordinated heme protein (HTHP), which was also immobilized electrostatically on a negatively charged SAM prior to electopolymerization [78]. The MIP-bound enzyme showed both DET and enzymatic substrate conversion. On the other hand, reports about MIPs for the hemeprotein hemoglobin (Hb) with the readout by DET are questionable since the formal potential reported is far too negative as compared with the value of the native protein [79,80].

### 2.2. Catalytically Active Analytes

#### 2.2.1. Enzymes and Enzyme-Labeled Analytes

For biocatalysts, MIP synthesis, template removal and the rebinding of the analyte can be quantified by evaluating the biocatalytic activity of the MIP sensor. The formation of a colored reaction product was indicated for trypsin, human Hb and cytochrome P450 BM3 using optical methods [81,82,83]. Furthermore, the indication of a redox-active reaction product at the electrode has been applied for the characterization of template removal and rebinding for electrochemical MIP sensors. This principle has been successfully applied for acetylcholinesterase (AChE), laccase, tyrosinase, butyrylcholinesterase (BuChE), and horseradish peroxidase (HRP) [84,85,86,87,88]. The indication of the surface activity of enzymes brought about measuring ranges of the respective MIP sensors in the picomolar to micromolar range. For the highly active BuChE, the enzymatic activity was measured via the anodic oxidation of thiocholine, which is the reaction product of butyrylthiocholine [87]. The sensor exhibited a linear response between 50 pM and 2 nM concentrations of BuChE with an LOD of 14.7 pM. 

**Figure 5:**
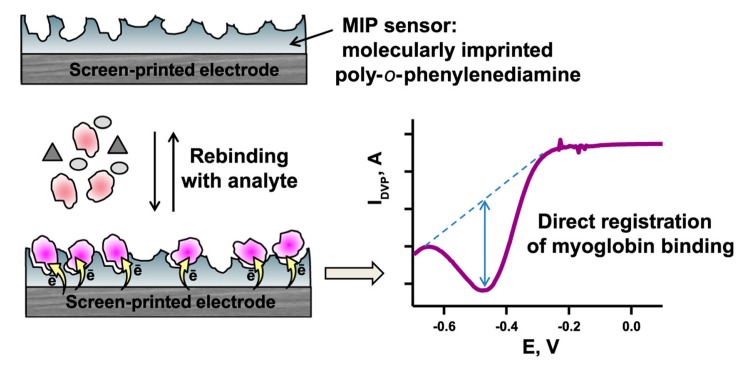
Schema of MIP preparation and the detection of myoglobin binding. Reprinted by permission from [90].

The surface activity sums up the substrate conversion by the enzyme molecules within the binding cavities and that of the nonspecifically adsorbed enzyme at the non-imprinted polymer surface. On the other hand, the generation of a catalytic current upon addition of the (co)substrate proves that the protein approaches the electrode surface with a “productive orientation” for DET. This approach was introduced by Reddy et al. for catalytic oxygen reduction in the presence of Hb [89] and was further adapted to myoglobin (Figure 5) [90] and bioelectrocatalytic peroxide reduction by MIP-bound HTHP [78]. 

**Figure 6:**
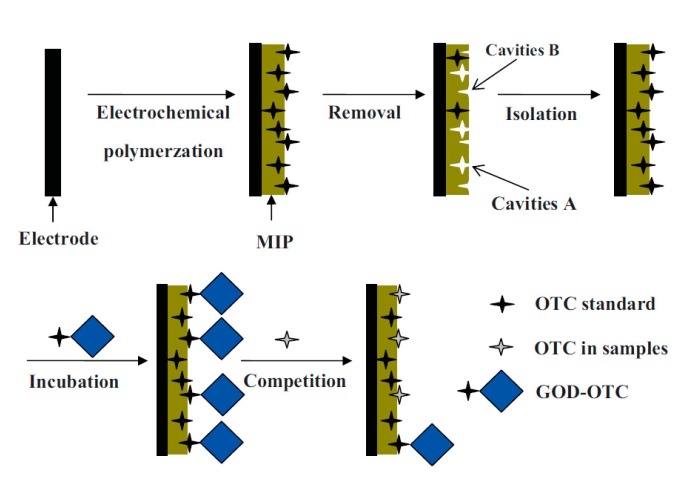
Schema of MIP preparation for oxytetracycline (OTC) and the principle of detection based on the glucose oxidase tracer (GOD–OTC). Reprinted by permission from [92].

Moreover, the coupling of MIPs with enzymes can enhance the analytical performance of biomimetic sensors. Signal generation by enzyme-labeled “tracers” has been applied analogously to competitive immunoassays in MIP sensors, e.g., for oxytetracycline (OTC) [91,92]. Glucose oxidase (GOD)-labeled OTC was used as tracer in a competitive assay format and the enzymatic activity was electrochemically evaluated (Figure 6). DPVs showed a concentration-dependent signal between 0 and 0.4 µM with an LOD of 0.33 nM [92].

The same group further enhanced the sensitivity of the MIP sensor for OTC by combining their approach with Prussian Blue (PB) [93], which reduces in neutral solutions hydrogen peroxide [94]. The MIP was prepared by electropolymerization in a solution containing OTC, polypyrrole, FeCl_3_, K_3_[Fe(CN)_6_] and KCl. It was demonstrated by DPVs that an increasing amount of OTC resulted in a decreased formation of H_2_O_2_, which is caused by the reduced amount of bound OTC-GOD. The sensor showed two linear ranges, i.e., 0–0.1 µM and 0.1–1 µM with an LOD of 230 fM.

An enzyme-labeled tracer has been further applied in an MIP sensor for the detection of streptomycin (STR), which has been used for the treatment of various bacterial infections [95]. The MIP sensor was prepared electrochemically on a gold electrode by copolymerizing aniline and o-phenylenediamine in the presence of STR. In comparison to the OTC-MIP, the measuring procedure does not include a separation step, but GOD–STR and STR mixtures were incubated for rebinding together. The hydrogen peroxide current was measured by DPV. The sensor had a linear response in the concentration range between 0.01 and 10 ng/mL STR and an LOD of 7.0 pg/mL was determined. The application of enzyme-labeled tracers in competitive formats allows for the extension to electroinactive analytes. However, the reagent costs are higher than for “direct” electrochemical sensors and the enzyme can hinder the interaction of the analyte with the MIP cavities and it may interact with the polymer surface.

#### 2.2.2. Catalytically Active MIPs

In addition to binding MIPs, which mimic the function of antibodies, enzyme mimics based on MIPs have also been developed. This field was pioneered by Wulff in 1972 [2]. By analogy with catalytically active antibodies, an analog of the transition state of the catalyzed reaction is applied as the template [96,97,98,99]. Efficient catalysis has been realized for splitting esters, carbonates and carbamates. These MIPs mimic catalysis by hydrolases. On the other hand, oxidoreductase mimics have been synthesized by integrating metal ions or metal complexes into the polymer matrix of MIPs [9,100]. As described for enzyme electrodes, the measuring signal is generated by the indication of an electroactive product or the consumption of a cosubstrate-like oxygen or peroxide.

Lakshmi et al. presented an electrochemical MIP sensor for catechol and dopamine using a Cu^2+^-containing layer of poly(N-phenylethylene diamine methacrylamide), which oxidized the template by ambient oxygen [101]. The polymer mimics the activity of the enzyme tyrosinase. The sensor’s response to catechol was linear up to 144 µM with an LOD of 228 nM. Furthermore, resorcinol, hydroquinone and serotonin did not interfere. However, for the regeneration of the sensor, reloading with Cu^2+^ was required. In another work, the enzyme nitroreductase was mimicked by a copper–melanin complex, which was used as the functional monomer. The MIP was prepared by electropolymerization on a chitosan capped AuNP-modified glassy carbon electrode in the presence of the drug metronidazole. The MIP sensor generated a concentration-dependent electrocatalytic current for the reduction of the nitro groups.

A mimic of the selenoenzyme glutathione peroxidase was built up by polymerizable amino acid derivatives as functional monomers and acryloyloxypropyl 3-hydroxypropyl telluride as the catalytic center [102]. The polymerization was performed in the presence of glutathione as a template. The MIP showed both specific substrate binding and peroxidase-like activity. 

**Figure 7:**
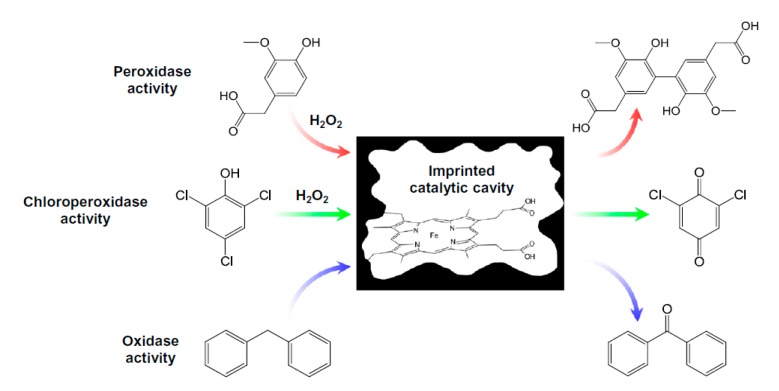
Hemin-based catalytically active MIPs. Reprinted by permission from [9].

The integration of redox-active groups of enzymes into the polymer scaffold is more straightforward than the application of simple metal complexes for the synthesis of enzyme mimics based on MIPs. Especially hemin, the active site of peroxidases and cytochrome P450 enzymes, has frequently been used (Figure 7). An MIP for homovalinic acid (HVA) which shows peroxidatic activity, was prepared by the copolymerization of hemin and HVA as a template [103]. It specifically bound to HVA and catalyzed its oxidation by hydrogen peroxide with a higher activity than towards (p-hydroxyphenyl)acetic acid and (p-hydroxyphenyl)propionic acid. Similar peroxidase mimics for the oxidation of p-aminophenol, serotonin or epinephrine were prepared by the bulk polymerization of methacrylic acid and integrated in a flow injection analysis (FIA) system with electrochemical detection [104,105,106]. Moreover, the FIA system has been applied for the measurement of serotonin in blood serum. In another work, chloroperoxidase-like activity towards 2,4,6-trichlorophenol (TCP) was demonstrated by Díaz-Díaz et al. for an MIP, which consisted of hemin as the catalytic center, TCP as the template and 4-viniylpyridine as a functional monomer [107]. Structurally similar substances did not influence the peroxide-dependent oxidation of TCP. Zhang et al. developed a hemin-containing dehalogenase-mimicking MIP, which indicated the formation of o-chlorobenzoquinone, the product of the peroxide-dependent reaction of 2,4-dichlorophenol, with an LOD of 1.6 µM [108]. Additionally, a peroxidase-mimicking MIP was prepared by using hemin as the catalytic center and 5-hydroxyindole-3-acetamide (5-HIAA) as a template [109]. It exhibited high activity towards the tumor marker 5-hydroxyindole-3-acetic acid. 

Phenazine methosulfate (PMS) was used as mimic of flavine adenine dinucleotide in an amperometric MIP sensor for fructosylvaline, which is the most important long-term parameter of diabetes [110]. The signal was generated by the electrochemical reoxidation of PMS which acts as a mediator.

The catalytic function in an enzyme-mimicking MIP has also been performed by metallic nanoparticles. Pt/Cu nanoparticles catalyzed the peroxide-dependent oxidation of MIP-bound puerarin in parallel with 3,3’,5,5’-tetramethylbenzidine (TMB) [111]. Lie et al. fabricated an MIP sensor for chlortoluron on the surface of magnetic NiO nanoparticles, which catalyzed the oxidation of H_2_O_2_ [112]. Chlortoluron was detected indirectly by the effect of target binding on the H_2_O_2_ oxidation current. 

Recently, the integration of an MIP structure into the pores of a catalytically active Cu-based MOF has been successfully demonstrated [113]. This approach realized the specific binding of the endogeneous disruptor tetrabromobisphenol A to the MIP with an imprinting factor of 7.6. The consumption of peroxide in the MOF-catalyzed oxidation was (colorimetric) indicated. In another work, the high potential of a catalytically active framework was exemplified for the hydrolysis of highly toxic organophosphorous compounds [114]. The porous aromatic framework was 14 times more effective than the natural enzyme organophosphorous hydrolase. 

The integration of the catalytically active MIPs in electrochemical sensors demonstrate that this approach has a high potential in respect to sensitivity, robustness and simple preparation.

### 2.3. Redox-Inactive Analytes

**Figure 8:**
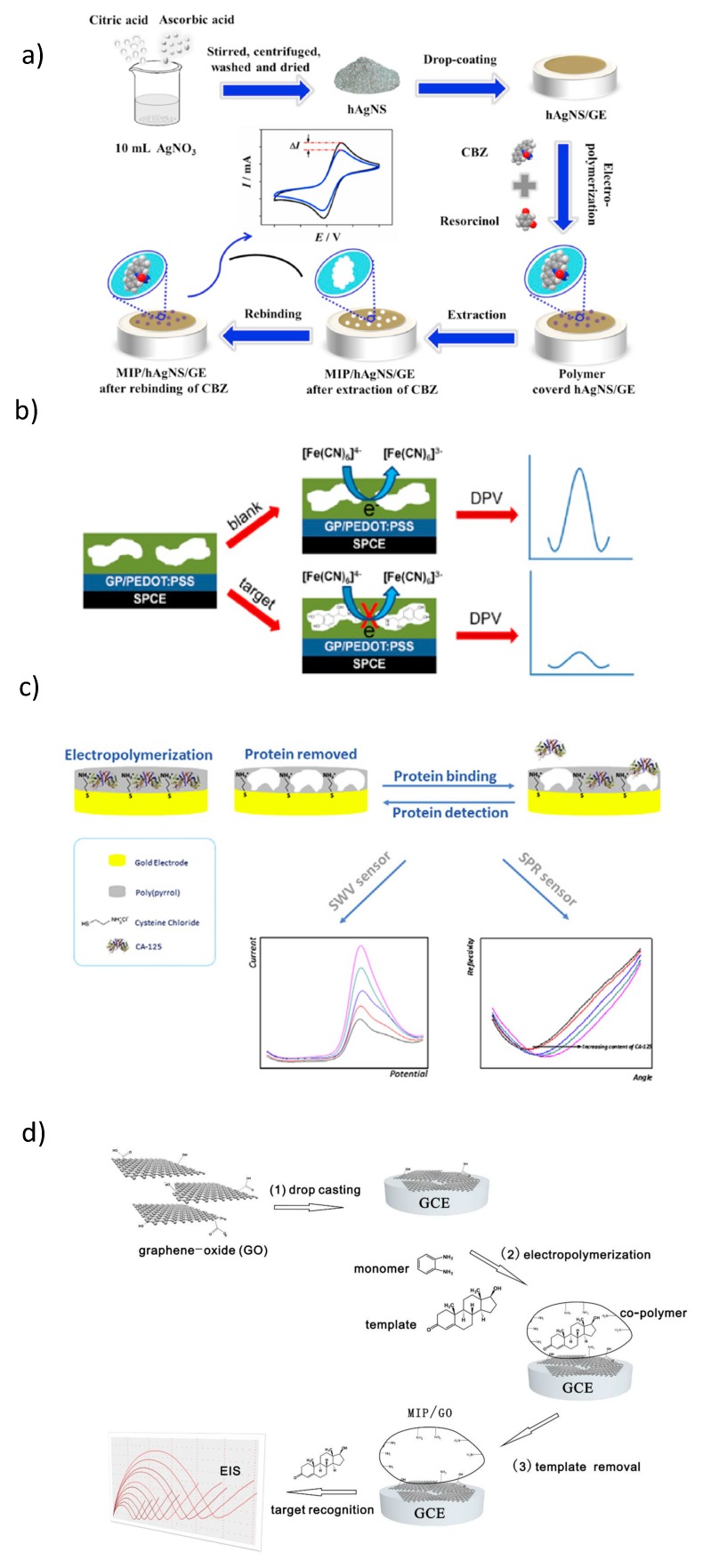
(**a**) Preparation of a carbamazepine MIP, reprinted by permission from [126]; (**b**) schematic representation of salbutamol MIP preparation and DPVs showing salbutamol binding to the MIP, reprinted by permission from [119]; (**c**) CA-125 MIP preparation by electropolymerization and SWV- and SPR-based measurements, reprinted by permission from [120] and (**d**) testosterone MIP preparation and EIS-based determination of testosterone, reprinted by permission from [121].

The most frequently applied method for the characterization of MIP sensors evaluates the diffusional permeability of the polymer layer to a redox marker, such as ferri/ferrocyanide, by cyclic voltammetry, differential pulse voltammetry, square wave voltammetry or electrochemical impedance spectroscopy (Figure 8) [49,56,60,115,116,117,118,119,120,121]. This approach is simple, cost-effective and highly sensitive. Furthermore, it provides characterization of each step of the MIP synthesis and the measurement of target rebinding to the MIP for low-molecular-weight targets, (bio)macromolecular and (nano)particles.

For low-molecular-weight molecules, the cavities after template removal have diameters comparable with that of the redox marker. Different mechanisms have been proposed for the influence of target binding on the current signal of the redox marker, including changes in the porosity of the MIP film or of the diffusion rate of the marker, doping–dedoping effects and changes in the electric double layer. The gate effect was, for the first time, described by Yoshimi et al. for a theophylline imprinted polymer, which was prepared by copolymerization of methacrylic acid and ethylene glycol dimethacrylate on an indium tin oxide electrode [122]. The model for macromolecules predicts that pores will be formed by the removal of the protein template in the tight MIP layer, which increase the permeation of the redox marker to the electrode surface. Rebinding of the target shrinks these pores, thus causing a concentration-dependent decrease in the permeation of the redox marker [37]. However, the exact mechanism of the “gate effect” is still not fully understood [122,123].

Metal organic frameworks have been applied in MIP sensors for the detection of redox-inactive analytes as well. Jiang et al. described an MIP sensor for the detection of aflatoxin B1 (AFB1), which was formed by electropolymerization of p-aminothiophenol-functionalized gold nanoparticles in the presence of the template [124]. The binding of AFB1 was indicated by linear sweep voltammetry of ferricyanide as a redox probe. The electron transfer rate increased when the concentration of AFB1 increased, due to a p-doping effect. The molecularly imprinted sensor exhibited a linear range, between 3.2 fM and 3.2 μM. Recently, a polypyrrole-based MIP for 17β-estradiol has been integrated into an MOF, which was modified with Prussian Blue [125]. Together with carbon nanotubes, PB increased the electrical conductivity, which resulted in an extremely high sensitivity with an LOD of 6.19 fM. 

**Figure 9:**
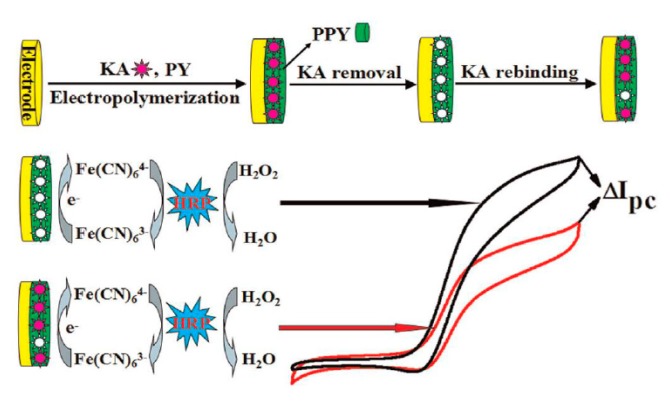
Preparation scheme of a kanamycin MIP using pyrrole (PPY) as a monomer, the signal is amplified by electro-enzymatic recycling of ferricyanide. Reprinted by permission from [127].

Enhancement of the sensitivity was further achieved by electro-enzymatic recycling for an MIP for kanamycin (Figure 9) [127]. The reduced redox marker, ferrocyanide, which was formed at the electrode, was subsequently reoxidized by horseradish peroxidase (HRP) in the presence of peroxide. The enzymatic recycling brought about an eight-fold higher signal and shifted the lower limit of detection by two orders of magnitude.

However, the analytical quality of the aforementioned approaches is problematic, since the rebinding of the target causes only small decreases in the large signal after template removal. Furthermore, the formation of “nonspecific” pores during template removal may influence the current signal. Different ionic strengths and/or pH during the rebinding and evaluation of the redox marker can falsify the measurement by structural changes of the polymer film. Nonspecific adsorption of surface-active constituents from the “real” sample may also influence the current. In addition, for the majority of redox marker-based MIP sensors, the signal of the redox marker is measured in a target-free solution, whereas rebinding occurs in ferricyanide-free solution. This procedure is, in principle, only applicable for MIP target systems with very low dissociation rates, which is a precondition and has been frequently ignored. Despite the inherent problems of the method, several papers describe MIP sensors for both small targets and macromolecules with lower limits of detection in the picomolar and even attomolar concentration range (Table 1). These publications evaluate either the relative or the absolute decrease in signal suppression in linear or semilogarithmic scales and frequently report two-phasic concentration dependencies without presenting a theoretical model for the binding.

## 3. Conclusions

Electrochemical methods allow not only the straightforward synthesis of MIPs, including polymer formation and template removal, but also the characterization of each step and a highly sensitive readout with simple devices. MIPs are highly effective for the suppression of interferences in the electrochemical indication of low-molecular-weight analytes by acting as shape-selective filters. The indication of the “gating effect” of target binding on a redox marker, which is widely used in electrochemical MIP sensors, has the disadvantage of generating an “indirect” measuring signal. It reflects not only the presence of the target but also changes in the polymer during the interaction with the sample. On the other hand, a highly specific approach is the evaluation of an electrocatalytic current for enzymes, since it couples enzymatic activity and DET in the cavities. Until now, this principle has only been demonstrated for heme proteins. The evaluation of catalytic currents may be applied in competitive assays, which use hemin and its derivatives as the catalytic component of the tracer. The integration of catalytically active MIPs into electrochemical sensors is promising in respect to robustness, stability and costs as compared with natural enzymes. 

Fully electronic MIP sensors are more common than sensors using spectroscopic methods, surface plasmon resonance or quartz crystal microbalance. In future, binding MIPs, so-called plastibodies, have the potential to substitute antibodies in affinity assays and sensors. MIP-based pocket-sized devices for critical analytes in medical emergencies and environmental supervision will adapt technologies from blood glucose meters, including self-powering by a fuel cell. Measurements by MIP sensors in real biological samples, e.g., blood, are still complicated by the presence of highly abundant proteins in the g/L region, e.g., serum albumin and immunoglobulin, while protein markers for cancer, diabetes or heart failure are typically in the mg/L to ng/L range. The required sensitivity has been reported in the literature for several MIP sensors based on the readout of redox markers (Table 1). However, the majority of tests have been carried out in “artificial” urine or spiked semi-synthetic plasma. Since MIP sensors represent only one “separation plate”, it is challenging to reach the required selectivity.

## Figures and Tables

**Table 1:** MIPs prepared by electropolymerization with electrochemical readout.

Template	Monomer	Electrode	Detection Method	Measuring Range and LOD	Reference
Tamoxifen	o-PD/Resorcinol	GCE	CV	1–100 nM	[60]
Lorazepam	Pyrrole	Sol-gel-AuNP-Graphite	SWV	0.2–2 nM and 2–20 nM; LOD: 0.09 nM	[128]
Kanamycin	o-PD	SWCNH-GCE	LSV	0.1–50 µM; LOD: 0.1 µM	[129]
2,4-dichlorophenol	o-PD	PDA-rGO-GCE	DPV	2–10 nM and 10–100 nM; LOD: 0.8 nM	[130]
p-Synephrine	Functionalized thiophene	Pt electrode	EIS	0.1–0.99 μM; LOD: 5.69 nM	[115]
Artemisinin	o-PD	Au electrode	CV, SWV	0.01–1.36 µM; LOD: 0.01 µM	[131]
Gemcitabine	PATP	AuNP-Au electrode	LSV	3.8 fM–38 nM; LOD: 3 fM	[132]
Sofosbuvir	PATP	N,S@GQDs-AuNP-PGE	DPV	0.1–40.0 × 10^−8^ M; LOD: 0.036 × 10^−8^ M	[133]
Sulfamethoxazole	o-PD	GCE	SWV	0.2–1.4 µM; LOD: 0.05 µM	[134]
Lidocaine	Resorcinol	Porous C-GCE	CV	0.2 pM–8 nM; LOD: 67 fM	[135]
Carbamazepine	Resorcinol	hAgNS-Au electrode	CV	8.0 × 10^−11^–6.0 × 10^−8^ M; LOD: 3.2 × 10^−11^ M	[126]
Cholesterol	PATP	AuNP-MWCNT-GCE	DPV	1 × 10^−13^–1 × 10^−9^ M; LOD: 3.3 × 10^−14^ M	[136]
Thyronamine	4-ABA	Carbon-SPE	SWV	up to 10 µM; LOD: 0.081 µM	[137]
17-β-estradiol	3,6-diamino-9-ethylcarbazole	GCE	EIS	1 aM–10 µM; LOD: 0.36 aM	[138]
Hexahydrofarnesol	o-PD	GCE	DPV	4.0 × 10^−8^–1.5 × 10^−7^ M and 1.5 × 10^−7^–1.5 × 10^−6^ M; LOD: 1.2 × 10^−8^ M	[139]
Nitrofurantoin	m-Dihydroxy-benzene/o-Aminophenol	GCE	EIS	0.001–0.05 µM and 0.1–1 µM; LOD: 0.3 nM	[140]
Dibutyl phthalate	Pyrrole	PGE	EIS	0.01–1.0 μM; LOD: 4.5 nM	[141]
Chlorpyrifos	Pyrrole	PGE	EIS	20–300 µg/L; LOD: 4.5 µg/L	[142]
Transferrin	Scopoletin	Au electrode	SWV	0.1–1 µM	[49]
HSA	Scopoletin	Au electrode	CV	20–100 mg/dm^3^; LOD: 3.7 mg/dm^3^	[143]
HSA	Bithiophene derivatives	Au electrode	DPV	12–300 pM; LOD: 0.25 pM	[144]
Ferritin	Phenol	Nanotube arrays	EIS	1 × 10^−12^ × 10^−7^ g/L	[116]
Annexin A3	Caffeic acid	Carbon-SPE	SWV	0.1–200 ng/mL; LOD: 0.095 ng/mL	[145]
Tyrosinase	o-PD	GCE	DPV	up to 50 nM; LOD: 3.97 nM	[86]
PSA	Pyrrole	Au-SPE	DPV	0.01–4 ng/mL LOD: 2 pg/mL	[146]
CA-125	Pyrrole	Au-SPE	SWV	0.01–500 U/mL; LOD: 0.01 U/mL	[120]
IL-1β	Eriochrome black T	EDOT-PATP-Carbon-SPE	EIS	60 pM–600 nM LOD: 1.5 pM	[147]
Protein A	3-aminophenol	SWCNT-SPE	EIS	LOD: 0.60 nM	[148]
HER2-ECD	Phenol	Au-SPE	DPV	10–70 ng/mL; LOD: 1.6 ng/L	[149]
cTnT	Aniline/Carboxylated aniline	rGO/C-SPE	DPV	0.02–0.09 ng/mL; LOD: 0.008 ng/mL	[150]

o-PD: *o*-Phenylenediamine; GCE: Glassy carbon electrode; AuNP: Gold nanoparticle; SWCNH: Single-walled carbon nanohorn; LSV: Linear sweep voltammetry; PDA-rGO: Polydopamine-reduced graphene oxide; 4-ABA: 4-Aminobenzoic acid; PATP: p-Aminothiophenol; N,S@GQDs: N,S co-doped graphene quantum dots; PGE: Pencil graphite electrode; hAgNS: Hollow silver nanospheres; SPE: Screen-printed electrode; MWCNT: Multi-walled carbon nanotubes; HSA: Human serum albumin; PSA: Prostate-specific antigen; CA-125: Carbohydrate antigen 125, IL-1β: Interleukin-1beta; EDOT: 3,4-Ethylenedioxythiophene; SWCNT: Single-walled carbon nanotubes; HER2-ECD: Human epidermal growth factor receptor 2; cTnT: Cardiac troponin T (cTnT).
